# Bacillus Calmette Guérin Osteomyelitis of the Proximal Tibia Extending to the Physis and Epiphysis in an Immunocompetent Toddler: A Case Report

**DOI:** 10.7759/cureus.78385

**Published:** 2025-02-02

**Authors:** Keisuke Takemoto, Shinji Kounami, Takashi Shimoe, Daisuke Fukui, Daisuke Tokuhara

**Affiliations:** 1 Department of Pediatrics, Wakayama Medical University, Wakayama City, JPN; 2 Department of Orthopedics, Wakayama Medical University, Wakayama City, JPN

**Keywords:** bacillus calmette-guérin vaccine, epiphysis, orthopedic complication, osteomyelitis, physis, subcutaneous abscess

## Abstract

Bacillus Calmette-Guérin (BCG) osteomyelitis is a very rare but serious complication of BCG immunization. Although most affected patients experience a good outcome, late orthopedic complications, such as discrepancy in leg length, are a concern. The impact of the extent of surgical intervention on minimizing orthopedic complications remains unclear, especially in patients with BCG osteomyelitis involving the physis. Here, we report the case of a 22-month-old immunocompetent boy who developed BCG osteomyelitis in the proximal metaphysis of the right tibia, which extended to the physis and epiphysis and was accompanied by a large subcutaneous abscess. In the current case, minimal curettage with anti-tuberculosis drugs, which can prevent damage to the physis and epiphysis, was not beneficial. Hence, complete curettage of the lesions was required. No disruption in bone growth was observed at the last follow-up evaluation performed 32 months after the first visit. Based on our experience, timely and sufficient curettage is essential to control the disease and prevent orthopedic complications in patients with BCG osteomyelitis involving the physis and epiphysis.

## Introduction

Bacillus Calmette-Guérin (BCG), a globally used live attenuated vaccine derived from *Mycobacterium bovis*, prevents severe disseminated *M. tuberculosis* infections in young children. The protective efficacy of BCG vaccination against serious tuberculosis infections in children, particularly those with tuberculosis meningitis, is 60-80% [1]. The adverse events caused by the BCG vaccine might be associated with the recipient's age, immune status, and vaccine strain. In Japan, from 2013 to the present, children are recommended to receive the BCG vaccine, using the BCG Tokyo-172 strain, between five and eight months of age [2].

The common but minor adverse events include local abscess formation and/or lymphadenitis, which often improves with close monitoring [3]. Albeit very rarely reported, osteomyelitis is a severe complication of BCG vaccination in both immunocompetent and immunodeficient children [4,5]. BCG osteomyelitis is believed to occur due to hematogenous infection from the site of inoculation. BCG osteomyelitis preferentially develops in the metaphyseal ends of long bones of the limbs, whereas axial bones are less commonly affected [6]. According to the latest Japanese epidemiologic data, the BCG osteomyelitis rate is 3.1/1 million persons aged <1 year [2]. Effective treatment is important to avoid sequelae, such as altered bone growth, in children with BCG osteomyelitis [7], whereas diagnosis is often delayed because of its rarity. There are no randomized controlled trials investigating the management of patients with BCG osteomyelitis, and the role of surgical intervention in minimizing altered bone growth remains controversial [8].

Here, we report the case of an immunocompetent 22-month-old boy who was diagnosed with BCG osteomyelitis of the proximal metaphysis of the right tibia, extending to the physis and epiphysis, accompanied by a large subcutaneous abscess.

## Case presentation

A 22-month-old boy exhibited an intermittent limping gait for a week and pyrexia for three days. He was able to walk normally during the examination, which did not reveal abnormal findings; therefore, in-home observation was advised. However, four weeks later, the limping gait had worsened, with a body temperature of 39°C and swelling and redness on the back of the right knee, as noted by his parents. He was admitted to our hospital with the suspicion of cellulitis or osteomyelitis. His medical history was normal, and he had no history of contact with individuals with tuberculosis infection. The patient had no family history of immunodeficiency. He had received all of the routine vaccinations without any complications. At six months of age, he was vaccinated with the BCG Tokyo-172 strain on the left upper arm in Japan.

**Figure 1:**
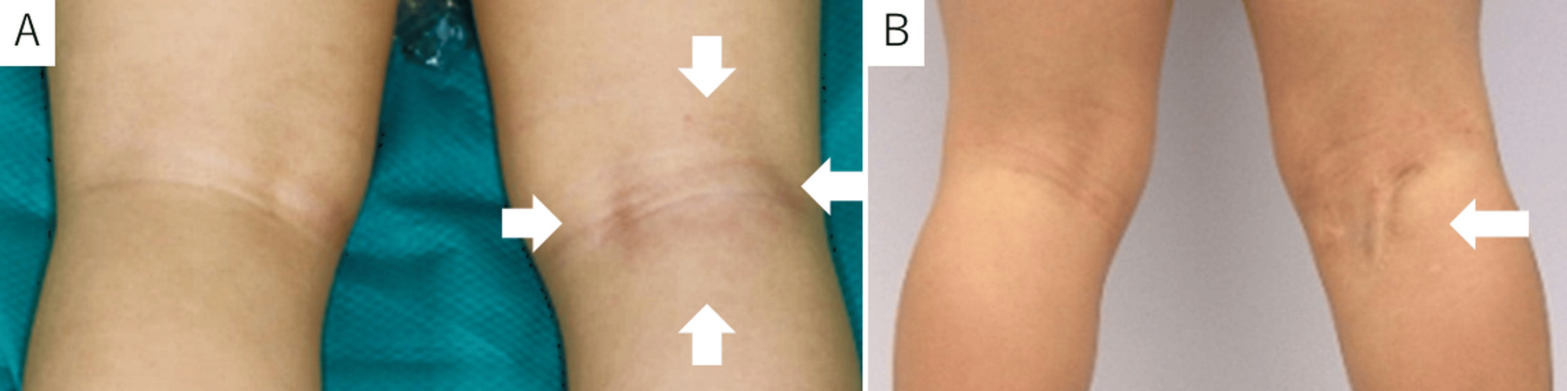
The back of the knee at admission and the final evaluation A: At admission, a 5 cm diameter swelling and skin redness were observed (arrow). B: At the final evaluation, the surgical wound gradually decreased in size (arrow).

Physical examination revealed swelling, erythema, and tenderness on the back of the right knee (Figure 1A).

The range of knee movement was restricted. Lymphadenopathy was absent. At admission, the laboratory test results were as follows: white blood cell count of 15,600/µL (neutrophils, 62%); C-reactive protein of 0.65 mg/dL; and erythrocyte sedimentation rate was 32 mm/h (see Table 1).

**Table 1:** Laboratory tests at admission

Laboratory tests (unit)	Results	Reference ranges
White blood cells (/µL)	15,600	4,200-12,000
Neutrophil (%)	62	33-55
C-reactive protein (mg/dl)	0.65	<0.15
Erythrocyte sedimentation rate (mm/h)	32	<15

**Figure 2:**
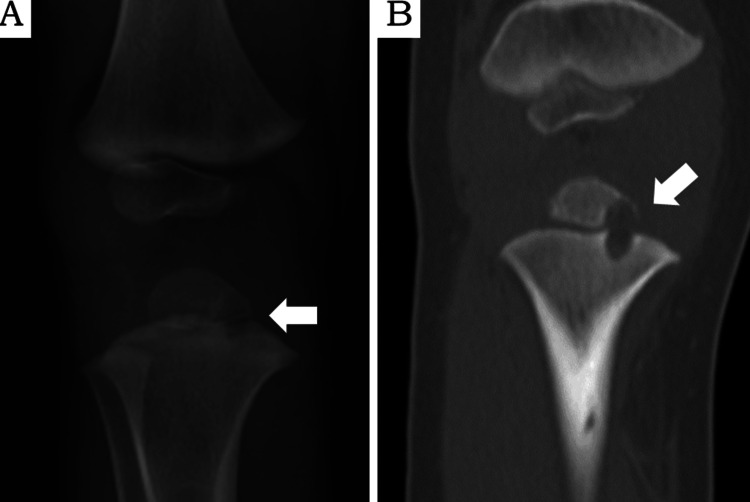
Radiographic images obtained at admission A: Plain radiograph of the right tibia showing a cystic lesion (arrow). B: Coronal computed tomography showing an osteolytic lesion in the metaphysis of the right tibia, extending to the physis and epiphysis.

Plain radiographs and computed tomography scans of the right knee revealed a lytic lesion in the proximal tibia, extending from the metaphysis to the epiphysis (Figure 2A-B).

**Figure 3:**
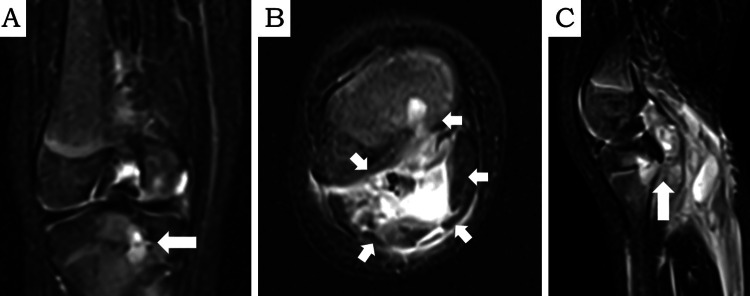
Fat-suppressed gadolinium-enhanced T2-weighted magnetic resonance imaging (MRI) obtained at admission A: Coronal view of the right tibia showing a high-intensity lesion in the bone marrow of the proximal tibial metaphysis and epiphysis (arrow). B: An axial MRI shows a subcutaneous abscess affected by peripheral enhancement by gadolinium. Abscess formation extending from the osteomyelitis lesion to the intermuscular and subcutaneous areas was observed (arrow). C: Sagittal MRI shows that the subcutaneous abscess is continuous with the osteomyelitis lesion (arrow).

Magnetic resonance imaging (MRI) revealed an intraosseous lesion and a large subcutaneous abscess contiguous with the metaphyseal lesion in the right tibia (Figure 3A-C).

The chest radiography result was normal, and no bones other than the right proximal tibia harbored lesions, determined with various imaging studies, including bone scintigraphy.

A yellowish transparent exudate was observed during the diagnostic first curettage of the subcutaneous abscess and metaphyseal lesion of the right tibia. The atheromatous tissue exiting the metaphyseal lesion was collected during curettage for pathologic evaluation. Treatment with cefazolin was started after admission. However, BCG osteomyelitis was suspected because of the lack of a strong inflammatory response. In addition to general bacterial culture, polymerase chain reaction (PCR) for *M. tuberculosis* complex was performed. The initially used PCR method can detect the *M. tuberculosis* complex, including the BCG strain. Nevertheless, further testing was required to determine whether it was a BCG strain. The *M. tuberculosis* complex was identified in the exudate and tissues with PCR two days after the first curettage. Pathologic examination of the atheromatous tissues revealed nonspecific granulation tissues containing multinuclear giant cells. Acid-fast-stained bacilli were not detected in the specimens. The tuberculin skin test and the interferon-gamma release assay were negative. These findings strongly supported the diagnosis of BCG osteomyelitis. *M. tuberculosis* complex was identified by culture, and multiplex PCR for *M. tuberculosis* complex revealed that the patient samples exhibited the pattern of the BCG Tokyo-172 strain. The patient's *M. tuberculosis* complex cultures were sensitive to all antituberculosis drugs except for pyrazinamide. The results matched the BCG Tokyo-172 strain. The gastric juice culture for *M. tuberculosis* complex had negative results. The ability of neutrophils to produce reactive oxygen species and the lymphocyte subsets were normal. The analysis of the genetic markers for Mendelian susceptibility to mycobacterial diseases, including IFNGR1, IFNGR2, IL12B, IL12RB1, IL12RB2, IL23R, STAT1, CYBB, IRF8, TYK2, RORC, JAK1, KBKG, and GATA2 revealed negative results.

**Figure 4:**
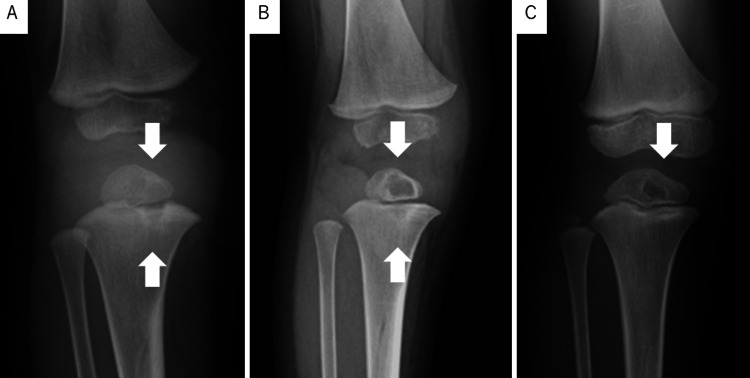
Bone radiography performed from just before the third curettage to the last evaluation A: Just before the third curettage, the lesion expanded (arrow). B: One month after the third curettage, the cavity caused by the curettage could be observed (arrow). C: At the last evaluation, there was no evidence of premature fusion of the epiphysis, and the cavity caused by the curettage shrunk (arrow).

Treatment with isoniazid, rifampicin, and ethambutol was initiated following the diagnosis of BCG osteomyelitis; however, the knee pain and fever persisted, and the subcutaneous lesion grew. Therefore, three weeks after the first surgery, a second procedure was performed to debride the subcutaneous tissues and curette the metaphyseal lesion in the right tibia under intraoperative fluoroscopy. No manipulations that could lead to injury to the physis and epiphysis were performed. After the surgery, the fever resolved, and the pain gradually improved. However, the surgical wound remained open, and the purulent exudate did not resolve despite the addition of streptomycin and levofloxacin to the chemotherapy regimen. Three months after the second surgery, the knee pain intensified in the setting of a body temperature of 38°C, requiring the third surgical intervention, which included the complete curettage of the lesions in the metaphysis, physis, and epiphysis under intraoperative fluoroscopy (Figure 4A-B).

The tibia was immobilized in a long leg cast for one month after the surgery, and all symptoms resolved thereafter. Ethambutol and streptomycin were replaced with clarithromycin and linezolid, in addition to isoniazid, rifampicin, and levofloxacin, which were continued for two months after the surgery. Thereafter, the regimen with isoniazid, rifampicin, and levofloxacin was continued for 12 months. The patient was treated with antituberculosis drugs for a total of 18 months after the diagnosis of BCG osteomyelitis. At the last evaluation, which was performed 32 months after the first visit, the patient had no signs of altered skeletal growth and no relapse of the lesions (Figure 4C). The surgical wound was gradually less noticeable (Figure 1B).

## Discussion

Although BCG osteomyelitis has a generally good prognosis [5], in a long-term follow-up study, Pöyhönen et al. reported that orthopedic sequelae were present in 13.8% of the patients with BCG osteomyelitis [7]. These complications might be related to inflammation at the site of infection and surgical procedures, and prompt diagnosis and proper intervention are critical to minimize complications in these patients.

In the present case, a positive PCR test for *M. tuberculosis* complex, easily available in tuberculosis treatment, was the first step in the diagnosis of BCG osteomyelitis. Although the identification of the BCG strain requires further evaluation, PCR is useful for the differential diagnosis of osteomyelitis in infants and young children. Furthermore, slightly elevated C-reactive protein levels and erythrocyte sedimentation rates compared with other types of bacterial osteomyelitis support the diagnosis of BCG osteomyelitis, as previously reported [9,10].

Currently, there are no standard guidelines for treatment approaches, particularly surgical treatment, for BCG osteomyelitis. A systematic review by Lin et al. revealed that orthopedic sequelae were more common in patients with BCG osteomyelitis undergoing surgical procedures [8], whereas the results of a large case series in Taiwan suggested that surgical procedures should be performed as a diagnostic procedure rather than as a treatment, considering late complications [6]. In the present case, treatment was initiated with minimally invasive surgical procedures to prevent damage to the physis, with the expectation that the antituberculosis drugs would be effective. There are no guidelines for the treatment of BCG osteomyelitis. Thus, we determined the appropriate type of drugs, changes or additions to the drugs, and the duration of treatment based on previous reports [6,8]. However, despite intensified antituberculosis drug treatment, the patient eventually required complete curettage, including all the lesions in the physis and epiphysis. In their meta-analysis, Ishimaru et al. suggested that patients with BCG osteomyelitis extending to the physis and epiphysis experienced a high recurrence rate of 55.6% and that early curettage of the entire lesion should be performed to prevent late complications, based on their experience [11]. Ohtera et al. have reported a case of BCG osteomyelitis that healed without late complication. In this study, sufficient curettage, including lesions in the physis and epiphysis, was required after a diagnostic curettage and enhanced antituberculosis drug treatment [12]. From their case series of epiphyseal osteomyelitis caused by Mycobacterium species, including BCG in Korea, Yoo et al. emphasized the importance of the early detection of physeal damage on MRI. Moreover, they suggested that sufficient curettage might be necessary if the treatment response to antituberculosis drugs is poor [13]. Based on our experience, it is critical to perform sufficient curettage in a timely manner while monitoring response to antituberculosis drugs.

In this case, an appropriate curettage was the most important factor in achieving a good outcome. However, another characteristic of this case is that the bone lesion was located on the periphery and formed a large subcutaneous abscess compared with the bone lesion. Although BCG osteomyelitis in long bones of limbs is rarely accompanied by subcutaneous abscess [14,15], the initial peripheral location of the osteomyelitis lesions and their progression to the formation of a subcutaneous abscess might have prevented the development of intramedullary lesions, contributing to the favorable outcome observed in the present case.

## Conclusions

BCG osteomyelitis is a very rare but serious complication of BCG immunization. Prompt diagnosis and intervention are critical to decrease the risk of complications. Although late orthopedic complications are a concern, there are no standard guidelines for treatment approaches, particularly surgical treatment, for BCG osteomyelitis. The impact of the extent of surgical intervention on minimizing orthopedic complications remains unclear, especially in patients with BCG osteomyelitis involving the physis. Based on our experience, timely and sufficient curettage is crucial to control disease and prevent orthopedic complications in patients with BCG osteomyelitis involving the physis and epiphysis.
